# Immunohistochemical expression of β-catenin, Ki67, CD3 and CD18 in canine colorectal adenomas and adenocarcinomas

**DOI:** 10.1186/s12917-021-02829-6

**Published:** 2021-03-12

**Authors:** Kristin M. V. Herstad, Gjermund Gunnes, Runa Rørtveit, Øyvor Kolbjørnsen, Linh Tran, Ellen Skancke

**Affiliations:** 1 Department of Companion Animal Clinical Sciences, Faculty of Veterinary Medicine, Norwegian University of Life Sciences (NMBU), Oslo, Norway; 2 Department of Preclinical Sciences and Pathology, Faculty of Veterinary Medicine, Norwegian University of Life Sciences (NMBU), Oslo, Norway; 3 Department of Animal Health, Norwegian Veterinary Institute, Section for Biohazard and Pathology, Oslo, Norway

**Keywords:** Canine, Colorectal adenoma and adenocarcinoma, Markers for tumor progression, Tumor-infiltrating immune cells

## Abstract

**Background:**

Inflammation is believed to influence human colorectal carcinogenesis and may have an impact on prognosis and survival. The mucosal immunophenotype in dogs with colorectal cancer is poorly described.

The aim of this study was to investigate whether the density, distribution and grade of tumor-infiltrating immune cells (TIIs) are different in normal colonic tissue vs benign stages (adenomas) and malignant stages (adenocarcinomas) of canine colorectal carcinogenesis, and thus, whether they can be considered as prognostic factors in dogs.

This retrospective case-control study was performed on formalin-fixed, paraffin-embedded tissue samples from dogs with histologically confirmed colorectal adenoma (*n* = 18) and adenocarcinoma (*n* = 13) collected from archived samples. The samples had been collected by colonoscopy, surgery or during postmortem examination. Healthy colonic tissue obtained post mortem from dogs euthanized for reasons not involving the gastrointestinal tract served as control tissue (*n* = 9).

**Results:**

The tumor samples had significantly lower numbers of CD3+ T-cells in the epithelium compared to controls (adenocarcinoma vs control, Kruskal-Wallis test, *p* = 0.0004, and adenoma vs control, *p* = 0.002). Adenomas had a significantly lower number of CD18+ cells in the lamina propria, compared to control samples (Kruskal-Wallis test, *p* = 0.008). Colonic samples from control dogs had uniform staining of β-catenin along the cell membrane of epithelial cells. Compared to normal colonic cells, the expression levels of cytoplasmic β-catenin were significantly higher in adenomas and adenocarcinomas (adenoma vs control Kruskal-Wallis test, *p* = 0.004, and adenocarcinoma vs control, *p* = 0.002). None of the control samples showed positive staining of β-catenin in the nucleus of colonic cells. In contrast, adenocarcinomas and adenomas showed moderate to strong staining of the cell nucleus. The nuclear β-catenin expression (signal strength and distribution) was significantly higher in adenomas compared to adenocarcinomas (Kruskal-Wallis test, *p* < 0.05).

**Conclusions:**

β-catenin and Ki67 were not useful markers for demonstrating tumor progression from adenomas to adenocarcinomas. The lower presence of CD18 and CD3+ cells in colorectal tumors compared to controls indicates a reduced presence of histiocytes and T-cells, which may have implications for the pathogenesis and progression of colorectal cancer in dogs.

**Supplementary Information:**

The online version contains supplementary material available at 10.1186/s12917-021-02829-6.

## Background

Colorectal cancer (CRC) is one of the most common types of cancer in humans [1]. In contrast to humans, CRC is rarely diagnosed in dogs, albeit more frequently than in other mammals [2, 3]. The disease is associated with serious clinical signs and has a poor prognosis due to local recurrence and metastases in humans [4, 5] and dogs [6–8]. Sporadic CRC in humans is believed to originate from adenomas through a process of multiple genetic and molecular events [9]. Studies indicate that a similar process occurs in canine colorectal carcinogenesis [10–14].

Colonic stem cells are located at the bottom of the colonic crypt where they proliferate and migrate towards the top, resulting in mature cells without the capacity to divide. This proliferation process is tightly regulated through the wingless-related integration site (Wnt) signalling pathway and involves the protein β-catenin [15]. Mutation of β-catenin is a key factor in CRC tumorigenesis, as described in humans [15] and dogs [10, 16, 17]. A failure in this mechanism results in colonic cells with increased proliferation capacity. Cellular proliferation in tumorigenesis may be evaluated by Ki67 [18]. The Ki67-protein is only present during active phases of the cell cycle; thus, expression of this antigen indicates cell-growth [19].

Inflammatory cells have been proposed to play a role in colorectal carcinogenesis in humans [20, 21], and chronic inflammation such as ulcerative colitis may progress to cancer [22]. In humans, inflammatory cells infiltrating colorectal adenoma and adenocarcinoma may influence the tumour’s capability to proliferate and metastasize [23]. The presence of tumor-infiltrating CD3+ T-lymphocytes in human CRC correlates with prognosis, as patients with high tumor infiltration have longer survival times than those with poorly infiltrated tumors [24]. CD18 is a panleucocyte marker, often used to characterize histiocytic cells, including monocytes and macrophages [25]. These macrophages, referred to as tumor-associated macrophages (TAMs), contribute to metastases in human colorectal carcinogenesis [26].

In Japan, miniature dachshunds seem to be predisposed to inflammatory polyps which may develop into adenoma and adenocarcinoma [17, 27, 28]. However, in these studies, TIIs were only described in inflammatory polyps and not in adenomas and adenocarcinomas [17, 28]. To the best of the authors’ knowledge, tumor-infiltrating immune-cells (TIIs) in canine CRC have not previously been characterized. In this work, we characterized and quantified the infiltration of immune cells in canine colorectal adenoma, adenocarcinoma, and normal colonic tissue using the antigens CD18 and CD3 labelling histiocytes and T-cells, respectively. Evaluation of tumor progression was performed using the antigens β-catenin and Ki67.

## Results

No significant difference in breed, age and gender were noted among dogs with colorectal adenoma, adenocarcinoma and control dogs (Kruskal-Wallis test, *p* > 0.1).

For the dogs with tumors, the following breeds were represented with ≥3 individuals: German Shepherd (*n* = 3) and English Setter (*n* = 3). The control dogs consisted of various breeds of which each was represented only once in the study material.

Information regarding tumor location was available in 22 dogs. The tumors were located in the rectum in 18 dogs, and in the colon in four. The localization of the remaining nine tumors was not specified (Table 1).

**Table 1:** Overview of dogs and samples

Dog no.	Breed	Gender	Age (y)	Diagnosis^a^	Tumor location (C/R)	Method of sampling	Treatment
1	German shepherd	M	9	Adenocarcinoma	R	Surgery	Surgery
2	Irish Setter	F	10	Adenocarcinoma	R	Colonoscopy	Surgery
3	Shetland sheepdog	M	14	Adenocarcinoma	C	Post mortem	Meloxicam
4	English springer spaniel	M	8	Adenocarcinoma	R	Colonoscopy	Piroxicam
5	Tibetan spaniel	M/N	10	Adenocarcinoma	R	Postmortem	Meloxicam
6	UN	M	10	Adenocarcinoma	UN	UN	UN
7	Doberman Pinscher	M	7	Adenocarcinoma	UN	UN	UN
8	Great Dane	F/N	6	Adenocarcinoma	UN	UN	UN
9	UN	M/N	9	Adenocarcinoma	UN	UN	UN
10	UN	F	8	Adenocarcinoma	R	UN	UN
11	Longhaired Collie	M	13	Adenocarcinoma	R	UN	UN
12	Bernese mountain dog	M	5	Adenocarcinoma	R	UN	UN
13	Flatcoated Retriever	F	9	Adenocarcinoma	UN	UN	UN
14	German Shepherd	F	9	Adenoma	UN	UN	UN
15	Irish Setter	M	6	Adenoma	UN	UN	UN
16	English Setter	M	8	Adenoma	UN	UN	UN
17	Mixed breed	M	10	Adenoma	R	Surgery	UN
18	German Shepherd	M	4	Adenoma	R	Surgery	Surgery
19	Staffordshire Bullterrier	M	8	Adenoma	R	Surgery	Surgery
20	Papillon	M	10	Adenoma	R	Surgery	Surgery
21	Collie Shorthaired	M	3	Adenoma	R	Surgery	Surgery
22	Norwegian Lundehund	M	7	Adenoma	R	Colonoscopy	Surgery
23	Cocker Spaniel	F	10	Adenoma	C	Colonoscopy	no
24	Golden Retriever	M	2	Adenoma	R	Surgery	Surgery
25	Bichon Havanais	M	5	Adenoma	R	Colonoscopy	Surgery
26	English Setter	M	11	Adenoma	R	Surgery	Surgery
27	Gordon Setter	F	10	Adenoma	R	Surgery	Surgery
28	Great Dane	M	10	Adenoma	C	Colonoscopy	Piroxicam
29	Cocker Spaniel	M	12	Adenoma	C	Colonoscopy	no
30	Border Collie	F	12	Adenoma	R	Colonoscopy	Surgery
31	English Setter	F	8	Adenoma	UN	UN	UN
32	West Highland White Terrier	M	15	Respiratory distress	NA	Post mortem	NA
33	Miniature Pinscher	F	12	Lung tumor	NA	Post mortem	NA
34	Staffordshire Bullterrier	F	13	General weakness	NA	Post mortem	NA
35	French Bulldog	M	3	Intervertebral disk hernia	NA	Post mortem	NA
36	Alaskan Malamute	F	7	Polyneuropathy	NA	Post mortem	NA
37	French Bulldog	F	3	Degenerative disk disease	NA	Post mortem	NA
38	Collie, Longhair	M	UN	Epilepsy	NA	Post mortem	NA
39	Pug	M	5	Urolithiasis	NA	Post mortem	NA
40	Chihuahua	M/N	3	Multiple fractures, RTA	NA	Post mortem	NA

^a^The diagnosis was not determined for all control dogs; thus symptoms/syndromes are described in some of the cases

*UN* Unknown

*NA* Not applicable

*RTA* Road traffic accident

**Fig. 1:**
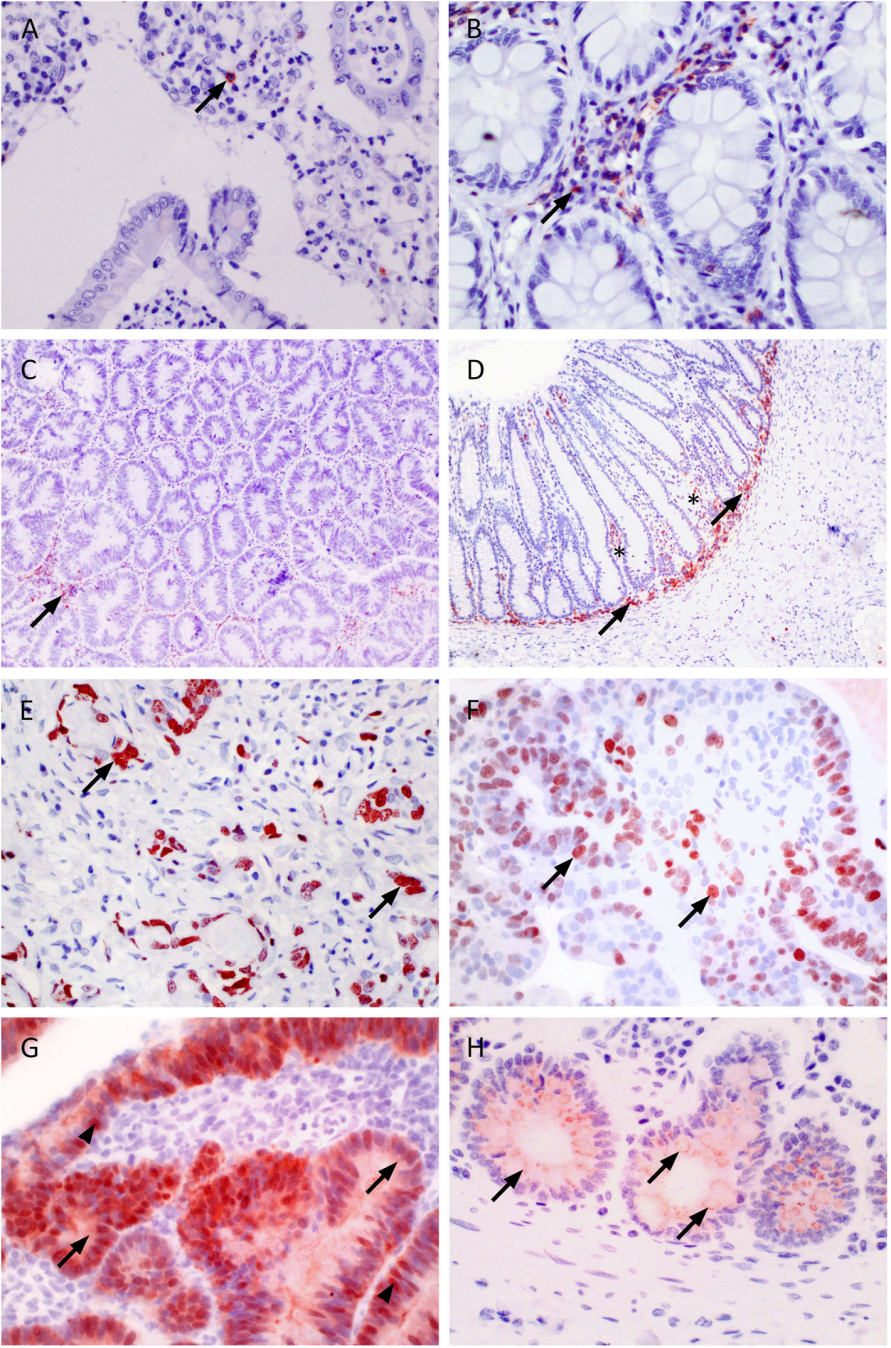
Immunohistochemistry. **a**. CD3, adenocarcinoma. A small number of positive cells were found in the lamina propria (arrow). 40x. **b**. CD3, normal colon. Scattered cells and clusters of cells were seen in the lamina propria (arrow) and the epithelium. 40x. **c**. CD18, adenocarcinoma. Fewer positive cells (arrow) and weaker signal than in the control. 10x. **d**. CD18, control. Characteristic distribution of positive cells, scattered in the lamina propria (*) and concentrated below the crypt epithelium (arrows). 10x. **e**. Ki-67, adenocarcinoma. Numerous cells have strong, nuclear staining (arrows). 40x. **f**. Ki67, adenoma. Numerous cells have strong, nuclear staining (arrows). 40x. **g**. β-catenin, adenocarcinoma. Strong staining of neoplastic epithelial cells. Both cytoplasmic (arrows) and nuclear (arrowheads) staining are evident. 40x. **h**. β-catenin, crypt epithelium from control. Moderate granular staining of cytoplasm only. The signal seems to partially follow the contour of the cell membrane (arrows). 40x

The number of CD3+ cells in the epithelium was significantly lower in adenomas (*n* = 18) and adenocarcinomas (*n* = 13) compared to control samples (*n* = 6) [Kruskal-Wallis test, adenomas vs controls, median 0 (IQR, 0–0) vs 1.5 (1–2), *p* = 0.002 and adenocarcinomas vs controls, 0 (0–0) vs 1.5 (1–2), *p* = 0.0004]. No difference was detected in lamina propria CD3+ cell-numbers between tumor samples and control samples (Kruskal-Wallis test, *p* > 0.1, Fig. 1a and b).

The adenoma samples (*n* = 18) had a significantly lower expression of CD18 positive cells in the lamina propria, compared to control samples (*n* = 8) [Kruskal-Wallis test, median 1 (IQR, 0–1.5) vs 2.25 (1.63–3), *p* = 0.008]. Adenocarcinomas (*n* = 12) had a significantly higher presence of CD18 positive cells when compared with adenomas (*n* = 18) [Kruskal-Wallis test, 1.75 (1–2) vs 1 (0–1.5), *p* = 0.05, Fig. 1c and d]. Although control samples had higher CD18 expression than tumor samples, this difference was not significant when comparing adenocarcinoma samples with control samples [Kruskal-Wallis test, 1.75 (1–2) vs 2.3 (1.7–3) *p* > 0.1].

The Ki67 positive cells showed a strong signal in adenomas (*n* = 14) and adenocarcinomas (*n* = 13), but no significant difference was detected in expression levels between these two tumor stages (Wilcoxon *p* > 0.05, Fig. 1e and f). None of the control samples expressed Ki67; thus, the IHC scoring of Ki67 was only reported in tumor samples.

Colonic samples from control dogs had uniform staining of β-catenin along the cell membrane of epithelial cells. Compared to normal colonic tissue, the expression levels of cytoplasmic β-catenin were significantly higher in adenomas and adenocarcinomas (Kruskal-Wallis test, adenomas vs controls, *p* = 0.004 and adenocarcinomas vs controls, *p* = 0.002, Table 2 and Fig. 1g and h).

**Table 2:** IHC score of β-catenin in tumors and controls (median, IQR and p-value)

	*P*-value β-catenin cytoplasma^1^	*P*-value β-catenin signal strength^2^	*P*-value β-catenin distribution^3^
Adenoma vs controls	*P* = 0.044 2.25 (1.5–3) vs 1 (1–1.75)	*P* < 0.0001 2.5 (2–3) vs 0 (0–0)	*P* = 0.03 1 (1–1.5) vs 0 (0–0)
Adenocarcinoma vs controls	*P* = 0.002 2 (2–3) vs 1 (1–1.75)	*P* = 0.02 0.25 (0–1.88) vs 0 (0–0)	*P* = 0.02 0.25 (0–1) vs 0 (0–0)
Adenoma vs adenocarcinomas	*P* > 0.5 2.25 (1.5–3) vs 2 (2–3)	*P* = 0.005 2.5 (2–3) vs 0.25 (0–1.88)	*P* = 0.03 1 (1–1.5) vs 0.25 (0–1)

^1^ Adenocarcinoma (*n* = 12), Adenoma (*n* = 18), Controls (*n* = 8)

^2^ Adenocarcinoma (*n* = 12), Adenoma (*n* = 16), Controls (*n* = 9)

^3^ Adenocarcinoma (*n* = 12), Adenoma (*n* = 16), Controls (*n* = 9)

There was a significant difference in β-catenin expression (signal strength and distribution) when comparing tumor samples with controls (Kruskal-Wallis test, *p* < 0.05, Table 2). None of the control samples showed any positive staining of β-catenin in the nucleus of colonic cells. In contrast, 16/31 of the tumor samples had moderate or strong staining of the nucleus. The presence of nuclear β-catenin expression (signal strength and distribution) was significantly higher in adenomas compared to adenocarcinomas (Kruskal-Wallis test, *p* < 0.05, Table 2).

Results of the IHC-scores can be found in Additional file 1.

## Discussion

Colorectal carcinoma develops from clonal expansion of genetically altered cells in humans [9] and dogs [10–14]. Although these genetic changes in tumors are well described, there are also other lesser-known mechanisms involved. Specifically, infiltrative inflammatory cells contribute to tumor progression by promoting cancer growth by releasing proangiogenic or pro-metastatic mediators or suppressing growth through an anti-tumor immune response [29, 30].

To the best of our knowledge, this is the first study focusing on inflammation in canine colorectal carcinogenesis. We found fewer epithelial CD3+ cells, representing T-cells, in colorectal adenomas and adenocarcinomas compared to control samples. Moreover, in adenomas, expression levels of CD18, representing histiocytes, were lower than controls.

Previous studies in humans with CRC have found that high T-cell proliferation is advantageous and associated with increased survival [23, 24, 31]. This association is believed to be due to T-cells initiating a cell-mediated immune response against target antigens on cancerous cells [32]. The lower tumor T-cell infiltration in our work may indicate the opposite; a reduced immune defense against tumor progression. Another explanation for the lower number of TIIs may be a lower expression of tumor-associated antigens by cancerous cells resulting in an inadequate anti-tumor immune response, and subsequently in a lower number of T-cells migrating to the tumor site [33]. The role of T-cells in canine carcinogenesis is not well described. In dogs with nasal carcinomas, CD3+ cells were reduced compared to normal nasal tissue [34]. In dogs with mammary cancer, high infiltrations of T-cells were associated with a poorer prognosis [35] and another study of dogs with brain gliomas, found that T-cell infiltrations were higher in high-grade glioma vs low-grade glioma [33, 36]. Importantly, CD3+ cells are indiscriminate markers for T-cells and do not differentiate between different subpopulations, with potentially opposing functions [37, 38]. For example, CD8+ and CD4+ T-cells have cytotoxic and immunomodulating properties, respectively [23]. Another marker, FoxP3, represents T-regulatory (Treg) cells, the presence of which may inhibit an efficient immune defense against tumor development [39]. Studies in humans with colorectal cancer have found an association between a low CD3+/FoxP3 ratio and shortened survival [40]. Thus, in order to clarify the potential role of T-cells in canine CRC, the subpopulations of T-cells need to be characterized.

In the present study, we found that expression levels of CD18+ cells were lower in adenomas compared to controls, which may indicate a reduced number of tumor-associated macrophages (TAMs) in adenomas [26, 41]. Studies in dogs have found that high levels of TAMs are associated with metastases and a poor prognosis in dogs with mammary adenocarcinomas [42, 43] and hemangiosarcomas [44]. As the CD18+ cells were increased in adenocarcinomas relative to adenomas, this might imply that TAMs are associated with malignancy. However, since the highest level of CD18 was observed in control tissue in our work, a more complex malignancy pattern may exist, and characterization of markers specific for TAMs is necessary to clarify the role of TAMs in canine CRC.

In humans, inflammation dominated by lymphocytes and plasma cells is more dominant in CRC compared to normal colonic mucosa [45], and non-steroidal anti-inflammatory treatment seems to be effective [46]. Despite our results showing a low presence of T-cells and histiocytes in canine colorectal tumors, anti-inflammatory treatment is often used and may be effective. Dogs with rectal polyps of varying malignancy, with minor infiltration of inflammatory cells treated with piroxicam rectally, improved with regards to clinical signs and size of tumors [47, 48]. This indicates that suppressing inflammation in these cases may be advantageous despite a lack of histologically confirmed inflammation. However, the efficacy of treatment with non-steroidal anti-inflammatory drugs may also depend on whether the tumor expresses cyclooxygenase (COX) [49].

Results from our study confirm previous descriptions of increased β-catenin expression in the cytoplasm and nucleus of cancerous cells compared to healthy colonic cells [10, 16, 50]. One study showed that this protein might correlate with tumor stage [10]. However, in the present study, the magnitude of nuclear β-catenin staining did not appear to correlate with tumor stage, as the adenoma samples demonstrated stronger nuclear β-catenin staining than the adenocarcinomas [16]. However, we cannot exclude the possibility that subtle changes may not have been detected with light microscopy. Other methods such as electron microscopy may be more sensitive for this purpose. Nevertheless, the lack of consistency among studies may be explained by several factors, including different histopathological methods used for tumor classification [51, 52]. A recent canine study showed that adenocarcinoma may be divided into subtypes based on whether they show polypoid or non-polypoid grow patterns [53]. Moreover, these tumors may also consist of different subtypes based on genetic alterations, as demonstrated in human CRC [54]. However, classification methods based on genetic markers are lacking in dogs. As in humans, adenocarcinoma in dogs could also be divided into subtypes which may benefit the evaluation of inflammatory markers in colorectal carcinogenesis.

The dogs with colorectal adenoma and adenocarcinoma consisted of various breeds, including English Setter and German Shepherd, each represented by three individuals. The latter breed is overrepresented in previous case reports of dogs with colorectal adenoma and adenocarcinoma [6, 8, 12, 55]. In the majority of dogs in our study (18/31 dogs), tumors were present in the rectum, and only four dogs had tumors in the colon. Similarly, in a study of 78 dogs with colorectal adenocarcinomas, over 85% of dogs had rectal tumors [6].

One limitation of this study is the low number of dogs, particularly in the control group. Due to the use of archived samples, clinical data are incomplete; thus, precluding further analyses, including survival analyses. Furthermore, the markers used to characterize inflammation in this study, CD3 and CD18, are insufficient to provide a complete picture of the immunophenotype, including T-cell subpopulation, TAMs and cytokine profiles associated with these cells [56]. Frozen samples are necessary for immunophenotype analyses and were unfortunately not available in the present study. The scoring of IHC signals is also a factor that may have influenced our results. For the Ki67 tumor scoring, we used a semi-quantitative scoring approach based on a subjective assessment of the IHC signals. This approach could have been replaced by more standardized methods such as computerized image analyses, which have been used in other studies scoring Ki67 in canine epithelial tumors [18, 57].

Although the control dog group was heterogeneous, the ages represented were comparable to those of the dogs with tumors. Healthy young laboratory dogs are commonly used as controls [16]. As age affects the degree of gastrointestinal inflammation [58, 59], it may be more relevant to include older dogs as controls, as in our study.

Markers are needed to distinguish adenomas and adenocarcinomas and thus evaluate prognosis. In humans, inflammatory markers have been suggested as part of the classification of malignant tumors [60]. This methodology, called “immunoscoring” may also be useful to distinguish adenomas from adenocarcinomas in dogs, and future studies should aim to determine the types and magnitudeof immune cell infiltration in canine colorectal tumors.

## Conclusions

In this work, we found a decreased presence of CD3+ cells, representing T-cells, in adenomas and adenocarcinomas compared to controls, as well as reduced presence of CD18+ cells, representing histiocytes, in adenomas compared to controls. The lack of these inflammatory cells may have implications for tumor progression. Future studies are needed to further clarify the role of TIIs in the development of canine colorectal cancer.

## Methods

This retrospective case-control study was performed on archived formalin-fixed paraffin-embedded tissue samples, collected for clinical purposes and submitted to NMBU during the period from 1998 to 2015. Owner consent was obtained for the samples to be used for research.

Inclusion criteria for this study were colorectal tissue from dogs with histologically confirmed colorectal adenoma or adenocarcinoma. Eighteen adenomas and 13 adenocarcinomas were included. Colonic tissue for control purposes was collected at necropsy from dogs euthanized for reasons not involving the gastrointestinal tract (*n* = 9).

### Selection of cases and control dogs

For each case, information about the breed, gender, age, histopathological diagnosis, tumor localization, sampling technique and treatment were obtained from the clinical record (Table 1). The dogs with colorectal adenomas and adenocarcinomas were of various breeds represented by both genders and had a median age of 8 years [min-max, 2–14]). Colorectal mucosal samples were collected from these dogs during surgery (*n* = 9), colonoscopy (*n* = 8), or necropsy (*n* = 2), and in 12 cases by an unknown procedure (Table 1).

The control dogs that contributed the healthy colonic samples consisted of various breeds from both genders and had a median age of 8 years [3–15] (Table 1).

### Tissue samples

Tissue specimens were fixed in 4% neutral buffered formalin, processed routinely, embedded in paraffin wax, cut into 4 μm thick sections and stained with haematoxylin and eosin (HE).

The histopathological diagnoses were evaluated by a board-certified veterinary pathologist (GG), according to the guidelines for classification of canine colorectal adenomas and adenocarcinomas [52]. These guidelines suggest that tumors are classified as adenocarcinoma only if neoplastic cells invade the muscularis mucosa. Although some of the tumors contained cellular features strongly indicative of malignancy, they were still classified as adenomas if no invasion of the basal lamina was found [52].

#### Immunohistochemistry

The following antibodies were used; mouse anti-β-catenin (BD Biosciences, Franklin Lakes, New Jersey), rabbit-anti-CD3 (DAKO, A 0452 North America Inc., California), mouse anti-dog-CD18 (Leucocyte Antigen Laboratory, California) and anti-Ki67 (Abcam, cat no. ab15580, Cambridge).

The sections were heat treated for antigen retrieval by autoclaving at 121 °C for 15 min in 0,01 M citric acid pH 6.0 for CD3 and Ki67, and in the microwave in pH 6.6 Target Retrieval Solution (DAKO, Glostrup, Denmark) for CD18 and pH 9.1 tris-EDTA buffer for β-catenin.

Endogenous peroxidase activity was inhibited with blocking reagent for 10 min (DAKO Envision system-HRP AEC REF K 4009 for CD3 and 3.0% H2O2 in methanol for Ki67, β-catenin and CD18). Non-specific antigenic sites were blocked with 5% bovine serum albumin (BSA) in Tris-buffered saline (TBS) for CD3, 1% normal goat serum (Vector/Bioteam) in 5% BSA/TBS for Ki67, 2% BSA in TBS for β-catenin and 10% normal goat serum in PBS for CD18. The sections were incubated at room temperature with the following primary antibodies, dilutions and incubation times: rabbit anti-CD3 (1:100 in 2,5% BSA, 60 min), rabbit anti-Ki67 (1:1000 in 2,5% BSA/ TBS, 60 min), mouse anti-dog-CD18 (1:100 in 10% goat serum, 30 min) or mouse anti-ß-catenin (1:2500 in 1% BSA/TBS, 60 min). Sections were then incubated for 30 min with secondary antibody from the DAKO Envision-kit for CD3, CD18 and ß-catenin, and goat anti-rabbit (DAKO, E 432) diluted 1:50 with 2% normal goat serum for Ki67. The Ki67- sections were then incubated for 30 min with Elite -ABC- kit (VECTASTAIN PK-6100) at diluted 1:50 in TBS. Color was revealed for 10 to 15 min using DAKO Envision system-HRP AEC for CD3, CD18 and ß-catenin, and the substrate solution (IMMPACT AEC PEROXIDASE SUBSTRATE SK-4205) for Ki67. Between the various steps, the sections were rinsed thoroughly in TBS. Finally, the sections were counterstained with haematoxylin solution for 45 s and mounted. Negative control staining was performed by replacing the primary antibodies with non-immunized goat serum and showed no staining. Lymph node sections were used as positive control tissue for the CD18 and CD3 staining. A canine skin tumor with numerous mitoses served as a positive control for the Ki67 staining. For β-catenin, the sample tissue itself served as an internal positive control, as all samples contained normal intestinal epithelium, and the cell membranes of these cells were expected to be positive for β-catenin.

#### Evaluation of immunohistochemistry

The sections were reviewed in a blinded fashion by the pathologists and were analyzed subjectively. Two pathologists evaluated the IHC score individually and agreed on the final score (CD18 and β-catenin; GG and RR, Ki67 and CD3; GG and ØK). For all sections, the IHC score was determined by evaluating the entire specimen.

For β-catenin, the scoring scheme included the prevalence of cells with a positively stained nucleus, using the following grading system: 0: no cells with a positive nuclear staining, 1: < 1/3 of cells with a positive nuclear staining, 2: 1/3–2/3 of cells with a positive nuclear staining and 3: > 2/3 of cells with a positive nuclear staining. Furthermore, the β-catenin staining intensity in the cytoplasm and the nucleus was scored from 0 to 3 (no staining, weak staining, moderate staining and strong staining).

An absence of nuclear staining for Ki67 was considered negative for this antigen. Ki67 scoring was only evaluated in the epithelium.

CD3+ cells were defined by clearly stained cytoplasm in the epithelium and within cells in the lamina propria.

For CD3, Ki67 and CD18, a semi-quantitative scoring scheme based on the prevalence of positively stained cells was applied, using the following grading system: 0: no staining, 1: few positive cells, 2: a moderate number of positive cells, and 3: many positive cells throughout the examined tissue.

The scores of the two pathologists were averaged, resulting in one score for each variable. If the difference between the two pathologists deviated by more than one grade (8 out of 228 scores), the slides were reviewed and discussed, resulting in a final score.

### Statistical analysis

The difference in demographic factors and the IHC score between dogs with adenoma, adenocarcinoma and control dogs were analysed using non-parametric tests (Wilcoxon test and Kruskal-Wallis test) JMP 14 (SAS, USA). A *P*-value < 0.05 was considered significant for all statistical tests. The median values and interquartile ranges (IQR, 25–75%) are listed. As the staining for all the antigens was not successful in all samples, the number of samples included is stated for each statistical test.

## Supplementary Information

**Additional file 1.** IHC score of CD3, CD18, Ki67 and β-catenin in tumor- and control samples.

## Data Availability

The datasets used and/or analyzed during the current study are available from the corresponding author on reasonable request.

## Abbreviations

CRC: Colorectal cancer
TIIs: Tumor-infiltrating immune cells
CD: Cluster of differentiation
TAMs: Tumor-associated macrophages
COX: Cyclooxygenase
