# Supplementary material for: Immunohistochemical expression of β-catenin, Ki67, CD3 and CD18 in canine colorectal adenomas and adenocarcinomas
Source: BMC Vet Res. 2021 Mar 12;17:119. doi: 10.1186/s12917-021-02829-6 (PMC7953700; doi:10.1186/s12917-021-02829-6)
Supplement: Supplementary file 1 — Additional file 1. IHC score of CD3, CD18, Ki67 and β-catenin in tumor- and control samples. [file 12917_2021_2829_MOESM1_ESM.docx]

**Additional file 1.** IHC score of CD3, CD18, Ki67 and β-catenin in tumor- and control samples

| Dog no. | Diagnosis* | CD3 LP | CD3 EC | CD18 | β-catenin  cytoplasma | β-catenin  nucleus  signal strength | β-catenin  nucleus distribution | Ki67 EC |
| --- | --- | --- | --- | --- | --- | --- | --- | --- |
| 1 | Adenocarcinoma | 1 | 0 | 1 | 2 | 0 | 0 | 3 |
| 2 | Adenocarcinoma | 0 | 0 | 1 | 2 | 2 | 1 | 3 |
| 3 | Adenocarcinoma | 2 | 1 | 3 | 3 | 1 | 1 | 3 |
| 4 | Adenocarcinoma | 2 | 0 | 2 | - | - | - | 3 |
| 5 | Adenocarcinoma | 1 | 1 | 1 | 2 | 0 | 0 | 3 |
| 6 | Adenocarcinoma | 1 | 0 | 1 | 2 | 0 | 0 | 1 |
| 7 | Adenocarcinoma | 1 | 0 | 1 | 2 | 2 | 1 | 1 |
| 8 | Adenocarcinoma | 2 | 0 | 2 | 3 | 3 | 3 | 2 |
| 9 | Adenocarcinoma | 0 | 0 | 2 | 3 | 1 | 1 | 0 |
| 10 | Adenocarcinoma | 0 | 0 | 2 | 3 | 3 | 2 | 0 |
| 11 | Adenocarcinoma | 1 | 0 | 0 | 2 | 0 | 0 | 1 |
| 12 | Adenocarcinoma | 2 | 0 | 3 | 1 | 0 | 0 | 2 |
| 13 | Adenocarcinoma | 1 | 0 | 2 | 3 | 0 | 0 | 1 |
| 14 | Adenoma | 1 | 0 | 0 | 2 | 2 | 1 | - |
| 15 | Adenoma | 0 | 0 | 0 | 2 | 3 | 3 | 3 |
| 16 | Adenoma | 2 | 1 | 2 | 1 | 2 | 1 | 2 |
| 17 | Adenoma | 0 | 0 | 2 | 2 | 3 | 2 | 2 |
| 18 | Adenoma | 1 | 0 | 1 | - | - | - | 3 |
| 19 | Adenoma | 2 | 1 | 1 | 3 | - | 1 | 1 |
| 20 | Adenoma | 0 | 0 | 0 | 2 | 3 | 1 | 2 |
| 21 | Adenoma | 0 | 0 | 2 | 3 | 2 | 1 | 3 |
| 22 | Adenoma | 1 | 0 | 2 | 3 | 3 | 1 | 3 |
| 23 | Adenoma | 2 | 0 | 0 | - | - | - | 2 |
| 24 | Adenoma | 1 | 0 | 2 | 3 | 3 | - | 3 |
| 25 | Adenoma | 1 | 1 | 3 | 2 | 0 | 2 | 2 |
| 26 | Adenoma | 2 | 1 | 0 | 3 | 3 | 0 | 3 |
| 27 | Adenoma | 1 | 1 | 0 | 3 | 3 | 2 | 3 |
| 28 | Adenoma | 0 | 0 | 1 | 2 | 2 | 2 | - |
| 29 | Adenoma | 0 | 1 | 0 | 3 | 3 | 1 | - |
| 30 | Adenoma | 0 | 0 | 0 | 3 | 3 | 1 | - |
| 31 | Adenoma | 0 | 0 | 3 | 1 | 3 | 2 | 0 |
| 32 | Control | - | - | 3 | 2 | 0 | 0 | - |
| 33 | Control | - | - | 3 | 1 | 0 | 0 | - |
| 34 | Control | - | - | 3 | 1 | 0 | 0 | - |
| 35 | Control | 2 | 1 | 3 | 2 | 0 | 0 | - |
| 36 | Control | 1 | 1 | - | 1 | 0 | 0 | - |
| 37 | Control | 1 | 2 | 2 | 2 | 0 | 0 | - |
| 38 | Control | 1 | 1 | 2 | 1 | 0 | 0 | - |
| 39 | Control | 0 | 2 | 0 | 1 | 0 | 0 | - |
| 40 | Control | 1 | 2 | 2 | 1 | 0 | 0 | - |

LP. lamina propria. EC. epithelial compartment

Samples with unsuccessful results after IHC were not scored for the given antigen.
